# A novel prognostic model for malignant patients with Gram-negative bacteremia based on real-world research

**DOI:** 10.1038/s41598-022-15126-5

**Published:** 2022-07-08

**Authors:** Sujiao Ni, Pingyao Xu, Kaijiong Zhang, Haiming Zou, Huaichao Luo, Chang Liu, Yuping Li, Yan Li, Dongsheng Wang, Renfei Zhang, Ruiling Zu

**Affiliations:** 1 Department of Clinical Laboratory, School of Medicine, Sichuan Cancer Hospital & Institute, Sichuan Cancer Center, University of Electronic Science and Technology of China, Chengdu, Sichuan China; 2 Chengdu University of Traditional Chinese Medicine, Chengdu, Sichuan China; 3 Department of Clinical Laboratory, The Third Hospital of Mianyang (Sichuan Mental Health Center), Mianyang, Sichuan China

**Keywords:** Cancer, Microbiology, Risk factors

## Abstract

Gram-negative bacteremia (GNB) is a common complication in malignant patients. Identifying risk factors and developing a prognostic model for GNB might improve the survival rate. In this observational and real-world study, we retrospectively analyzed the risk factors and outcomes of GNB in malignant patients. Multivariable regression was used to identify risk factors for the incidence of GNB, while Cox regression analysis was performed to identify significant prognostic factors. A prognostic model was constructed based on Cox regression analysis and presented on a nomogram. ROC curves, calibration plots, and Kaplan–Meier analysis were used to estimate the model. It comprised 1004 malignant patients with Bloodstream infection (BSI) in the study cohort, 65.7% (N = 660) acquired GNB. Multivariate analysis showed gynecologic cancer, hepatobiliary cancer, and genitourinary cancer were independent risk factors related to the incidence of GNB. Cox regression analysis raised that shock, admission to ICU before infection, pulmonary infection, higher lymphocyte counts, and lower platelet counts were independent risk factors for overall survival (OS). The OS was significantly different between the two groups classified by optimal cut-off value (log-rank, *p* < 0.001). Above all, a nomogram was created based on the prognostic model, which was presented on a website freely. This real-world study was concentrated on the malignant patients with GNB and proved that shock, admission to ICU before infection, pulmonary infection, higher lymphocyte counts, and lower platelet counts were related to the death of these patients. And a prognostic model was constructed to estimate the risk score of mortality, further to reduce the risk of death.

The morbidity and mortality of cancers were increasing year by year. Due to progression and treatments of cancers, the malignant patients were easier to get immunocompromised, which was related to multi-infections, especially bloodstream infection (BSI)^1^. BSI is a serious bacterial infection that not only brings more health care costs but also increases the mortality of malignant patients^2^. Chinese CHINET (China Antimicrobial Surveillance Network) has shown that gram-negative bacilli was the main isolate cultured from blood in Chinese major hospitals^3^. A 20-years study from 45 nations also supported the results^4^.

As reported, the isolation rate of extended-spectrum beta-lactamase-producing *Enterobacteriaceae* (ESBL-PE) and carbapenem-resistant organisms were rising recently, correlated to stubborn infections^5^. The BSI caused by gram-negative bacilli progressed more severer and caused higher mortality than gram-positive organisms. Even though the GNB was widely researched, there was still a paucity of data regarding the clinical features and death risk factors in malignant patients^6,7^. For the BSI patients, APECHII and SOFA were widely used in the evaluation of infectious shock^8^, but limited for the complex calculation and subjective estimation. So a prognostic model was required to estimate the dead risk of the malignant patients with suspicious GNB-BSI more objectively and fastly.

The real-world study was widely suggested to provide medical evidence recently. It tended to perform a long-term evaluation based on quite a large sample and focus on outcome measures that were clinically meaningful^9^. In this observational and retrospective real-world study, a large real-world cohort was included to define the clinical features and death risk factors of malignant patients with GNB. Additionally, this study included laboratory features lacking in most other studies, but with the potential to estimate the incidence and mortality of GNB. So based on those features, we constructed a prognostic model to indicate the risk of death. This tool only used information that was available from the clinical cases and laboratories, which could be obtained quickly online via the web page. The objective of this study was to develop a tool that could assess the patients’ prognosis readily.

## Materials and methods

### Study population

The malignant patients who were suspicious of BSI and delivered blood culture samples from July 2012 to September 2020 in Sichuan cancer hospital were retrospectively enrolled in this research. This research included inpatients who were aged 16-year or older. The pathology of all malignant patients was diagnosed by the pathology department in Sichuan cancer hospital and based on the diagnostic guidelines. The Centers for Disease Control and Prevention (CDC) definition for nosocomial infections was used as a reference to diagnose the BSI and GNB^10^. The clinical condition including primary infection sites, com-morbidities, therapeutic tools, and laboratory results on the day blood culture samples delivered was reviewed and recorded. The culture results and survival conditions of all enrolled patients were also recorded. The patients with incomplete information and blood culture contamination results were excluded.

### Study design

**Figure 1:**
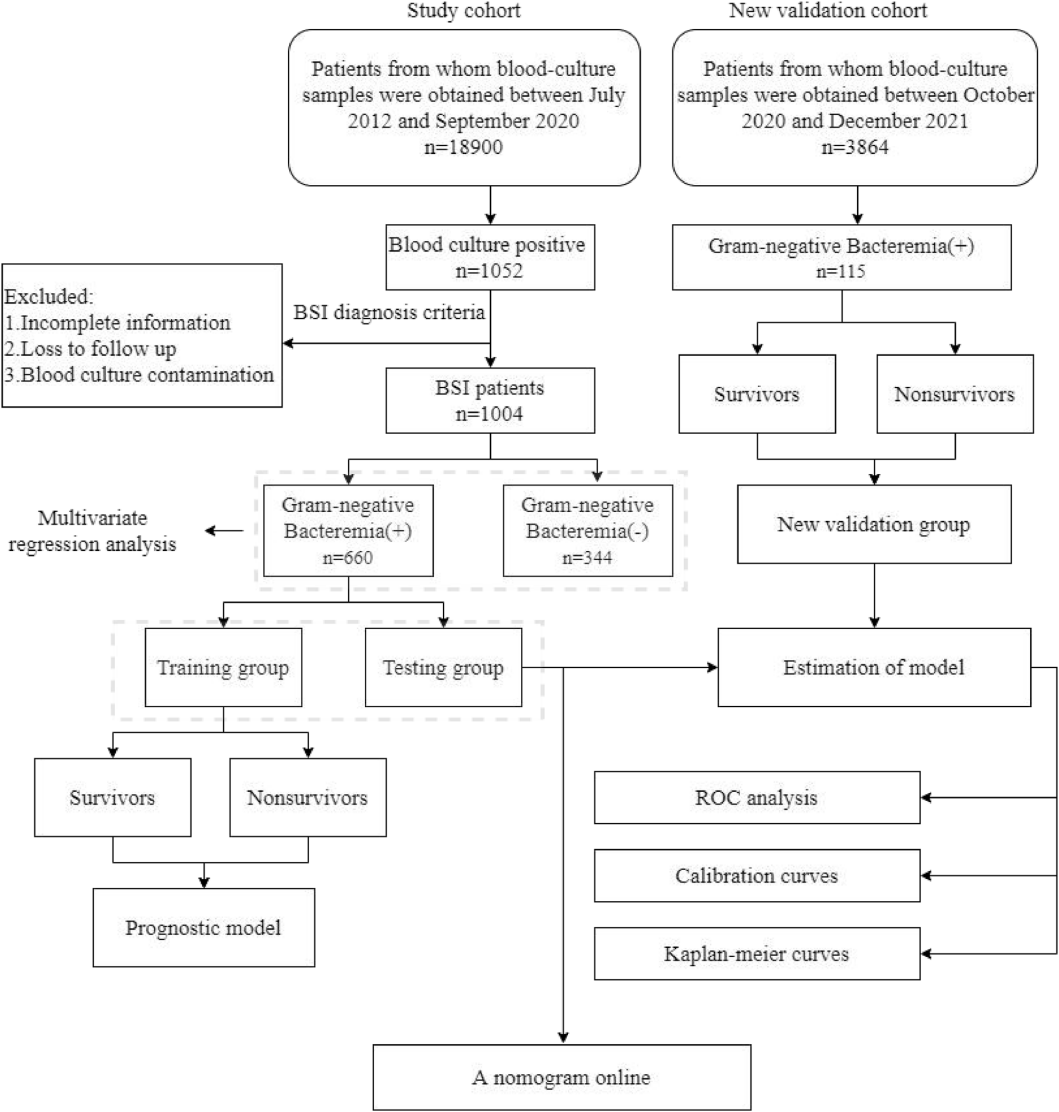
Study design. This research began with records of all patients whose blood-culture samples were obtained between July 2012 and September 2020. All participants were separated into training and testing cohort. And a new validation cohort were obtained between October 2020 and December 2021. A prognostic model was constructed using a training cohort estimated in testing and new validation cohorts. Finally, an online nomogram was generated based on the prognostic model.

The study design flow diagram was shown in Fig. 1. Patients whose blood-culture samples were obtained between July 2012 and September 2020 were collected for the study cohort. The number of malignant patients who underwent blood-culture tests was 18,900 (5.3% of 355,336 visits in the period of the time). The number of patients who had positive blood culture results was 1052. According to the isolation of blood culture, the enrolled patients were separated into Gram-negative bacilli infection (Gram-negative Bacteremia (+)) group and non-Gram-negative bacilli infection (Gram-negative Bacteremia (−)) group. Multivariable regression was used to identify risk factors for the incidence of GNB. According to the 30-days survival status, the patients in Gram-negative Bacteremia(+) group were separated into survivors and nonsurvivors groups. Cox regression analysis was performed to identify significant prognostic factors. And a prognostic model was constructed based on Cox regression.

New cohort validation data came from patients whose blood-culture samples were obtained between October 2020 and December 2021 in Sichuan Cancer hospital. The number of malignant patients who underwent blood-culture tests was 3864. 115 GNB positive patients were finally involved in a new validation cohort, and also according to the 30-days survival status, patients were separated into survivors and nonsurvivors groups.

The time ROC curves, calibration plot, and Kaplan–Meier analysis were used to estimate the model in testing group and new validation cohort. After all, a nomogram was created based on the prognostic model and presented on a website.

### Laboratory methods

The blood samples were collected before initiation of antibiotic treatment using BD BACTEC Standard Anaerobic and Aerobic medium (Becton Dickinson, Sparks, MD, USA), and then were cultured in BD BACTEC FX blood culture automated systems (Becton Dickinson, Sparks, MD, USA). The Sensititre ARIS 2X (Thermo Fisher Scientific, 81 Wyman Street, Waltham, UK) automatic susceptibility and identification system were used for bacterial identification and drug susceptibility testing. Disc diffusion test (Oxoid Ltd/Thermo Fisher Scientific, UK) was used to supplement Antibacterial susceptibility testing. Results Interpretation Reference to the guidelines of the Clinical and Laboratory Standards Institute(2017)^11^. Escherichia coli ATCC 25,922, and Pseudomonas aeruginosa ATCC 27,853 were used as internal quality control.

Procalcitonin (PCT) was measured using an automated immunofluorescent assay (Brahms KRYPTOR, Hennigsdorf, Germany). The normal PCT concentration was defined as < 0.10 ng/ml. C-reactive protein (CRP) levels were measured by nephelometry (Goldsite Diagnostics Inc, Shenzhen, China). The routine blood tests were measured by a blood routine analyzer (Mindray Medical International, Shenzhen, China). We used quality controls that were regularly checked by the National Center for Clinical Laboratory. All methods were carried out following relevant guidelines and regulations.

### Statistical analysis

Data management, statistical analyses and all the figures were conducted using R version 4.0.3. Clinical characteristics of the participants were summarized by median and inter-quartile range for continuous measures and counts with proportions for categorical features. The training and testing cohorts of GNB patients were selected by the random split-sample method (split ratio: 7:3)^12^. The statistical handle was performed with the Caret R package. The information including age, gender, underlying cancer, co-morbidities, infection status, primary infection site, and treatment status were compared using Chi-square and t test. Analyses of laboratory features were performed by the Kruskal–Wallis test, and the Mann–Whitney test was used for the two groups’ comparison. Then all information and laboratory features were added into multivariable logistic regression analyses to select the risk factors for the incidence of GNB-BSI infection^6,7,13^. The GNB associating variables with a *p*-value less than 0.05 were candidated for backward stepwise multivariate analysis with the Akaike information criterion (AIC) to investigate independent risk factors. According to regression results, the potential risk factors were performed in multivariate analyses to select the best-fit model. Multivariable time-to-event analysis was performed using Cox proportional hazards regression models to develop a nomogram using weighted estimators corresponding to each covariate derived from fitted Cox regression coefficients and estimates of variance. Survival curves were depicted using the Kaplan–Meier method and compared using the log-rank test. Cox regression analysis, the time ROC curves, calibration curve, and survival curves were completed using R. In all analyses, *P* < 0.05 was considered significant.

### Ethical approval and consent to participate

The study was approved by the  medical ethical committee of Sichuan Cancer Hospital (SCCHEC-02-2022-001), which waived the requirement for informed consent owing to the retrospective design of the study.

## Results

### Clinical characteristics

A total of 1119 patients were eligible for this study, which included 775 (69.3%) with GNB and 344 (30.7%) with other bacteremia. Based on a rule of thumb for sample size^12^, the sample size needed in this study was to have at least 10 outcome events per parameter estimating, and thus the total needed sample size was calculated at least 430 patients. So, all available data were used to maximize the power and generalizability of the results. While there were 660 patients with GNB and 344 patients with other bacteremia in the study cohort, the basic information of study cohort was presented in Table S1. All the GNB patients were divided into two groups which consisted of the training cohort (n = 459) and the internal testing cohort (n = 201). Then a new validation cohort including 115 patients with  GNB was collected.

The most seen underlying disease in these three groups was gynecologic cancer and upper gastrointestinal cancer. And the most common primary infection site of the three groups was blood, followed by pulmonary infection and urinary tract infection. The demographics and characteristics of all patients in different data sets were summarized in Table 1.

**Table 1:** Baseline demographics and characteristics of all patients in different data sets.

Characteristics	Training cohort (n = 459)	Testing cohort (n = 201)	New validation cohort (n = 115)
Age (median, IQR, years)	58.0 (49.0–65.5)	54 (48.0–66.0)	60.0 (54.0–68.0)
**Gender (n,%)**			
Male	206 (44.9%)	71 (54.9%)	63 (54.8%)
Female	253 (55.1%)	130 (45.1%)	52 (45.2%)
**Underlying disease (n,%)**			
Gynecologic cancer	147 (32.0%)	70 (17.2%)	32 (27.8%)
Upper gastrointestinal cancer	52 (11.3%)	19 (15.4%)	20 (17.4%)
Hepatobiliary cancer	48 (10.5%)	15 (4.1%)	18 (15.7%)
Genitourinary cancer	43 (9.4%)	17 (5.5%)	16 (13.9%)
Head and neck cancer	37 (8.1%)	14 (13.4%)	8 (7.0%)
Lung and bronchus cancer	35 (7.6%)	14 (14.5%)	2 (1.7%)
Breast cancer	22 (4.8%)	17 (6.4%)	3 (2.6%)
Other cancers	75 (16.3%)	35 (23.5%)	16 (13.9%)
**Primary infection(n,%)**			
Bloodstream	201 (47.8%)	102 (50.7%)	28 (24.3%)
Pulmonary	96 (20.9%)	31 (15.4%)	18 (15.7%)
Urinary tract	65 (14.2%)	32 (15.9%)	28 (24.3%)
Intraperitoneal infection	42 (9.2%)	20 (10.0%)	20 (17.4%)
Catheter related bloodstream infection	18 (3.9%)	5 (2.5%)	6 (5.2%)
Soft tissue	12 (2.6%)	5 (2.5%)	5 (4.3%)
Biliary tract	25 (5.4%)	10 (5.0%)	10 (8.7%)

*IQR* Interquartile range.

### Risk factors contributing to the incidence of GNB

As shown in Table S1, in the study cohort, the patients with gynecologic cancer, upper gastrointestinal cancer, and hepatobiliary cancer were more likely to get GNB. And the primary infection, such as pulmonary, urinary tract, and soft tissue might also lead to the GNB. Admission to ICU before infection, chronic obstructive pulmonary disease, and primary antibiotic exposure were also related to GNB. The analysis of laboratory features showed that higher PCT level (1.23, ng/ml, IQR (0.36–8.54), *P* < 0.05 and lower lymphocyte count level (0.42, *10^9^/L, IQR (0.22–0.73), *P* < 0.05) were related to GNB (Fig. S1).

**Figure 2:**
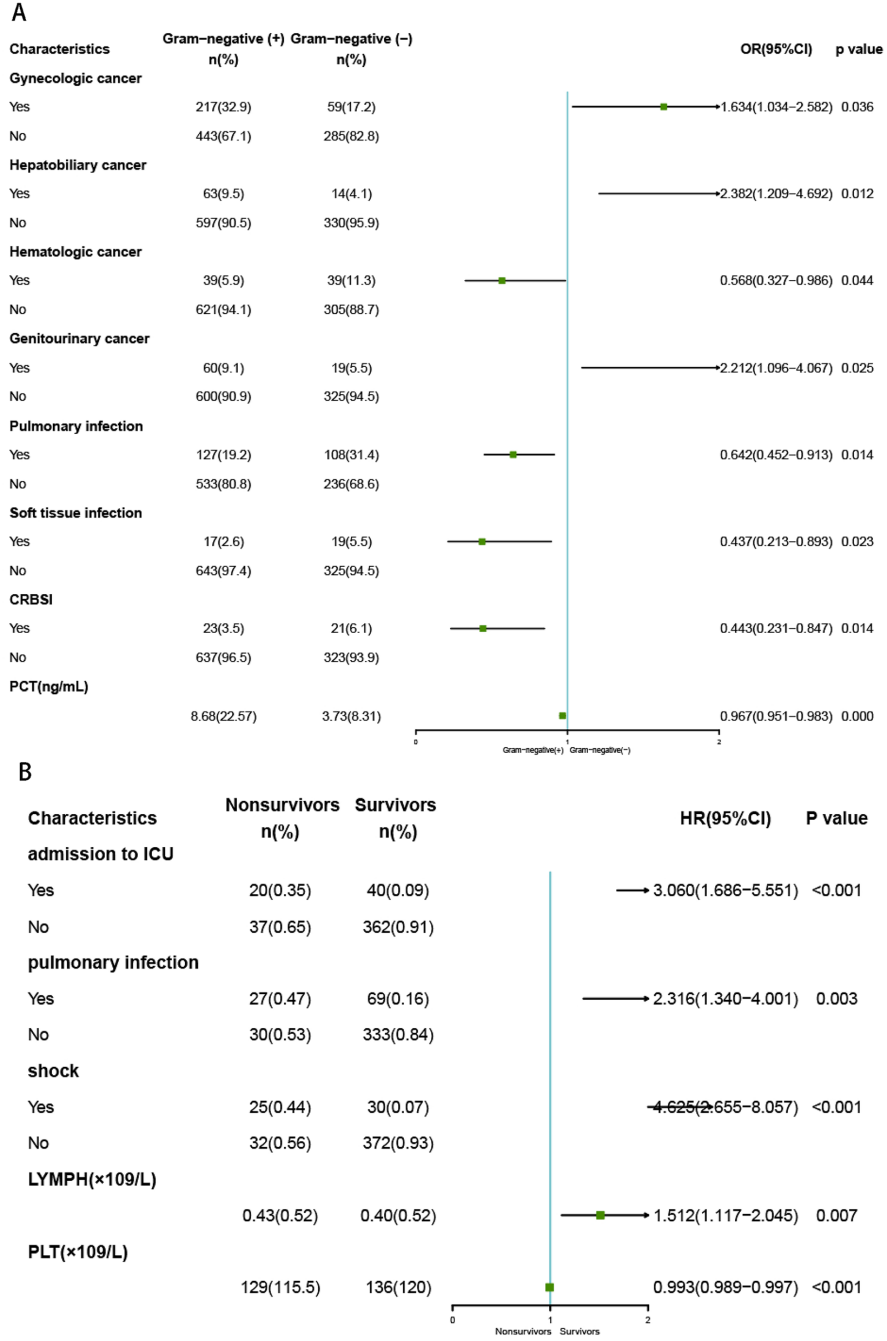
Forest plots showing the multivariate analysis results. Logarithmic odds ratios for GNB infection (**A**). Cox proportional hazards regression model for surviors in malignant patients with GNB (**B**).

The multivariate logistic regression analysis (Fig. 2A) showed that gynecologic cancer (OR = 1.634, *P* = 0.036), hepatobiliary cancer (OR = 2.382, *P* = 0.012), genitourinary cancer (OR = 2.212, *P* = 0.025) were independent risk factors for GNB. Hematologic cancer (OR = 0.568, *P* = 0.044), pulmonary infection (OR = 0.642, *P* = 0.014), catheter related bloodstream infection (OR = 0.443, *P* = 0.014), soft tissue infection (OR = 0.437, *P* = 0.023) and lower PCT (OR = 0.967, *P* = 0.000) had lower percentages of GNB. All the multivariate logistic regression results were shown in Table S2.

### Risk factors for 30-day mortality in patients with GNB

The patients with GNB from the study cohort were classified into survivor and non-survivor groups according to the 30-day survival status. The overall 30-day mortality rate in GNB patients was 12.27% (81 of 660). Multiple factors were associated with poor prognostic, which were shown in Table S3. According to the multivariate logistic regression analysis, shock, admission to ICU before infection, pulmonary infection, nasogastic tube, and lower platelet counts (PLT) were related to the poor prognostic (Fig. S2).

### Prognostic model constructed using Cox-regression

The training and testing cohorts of GNB patients consisted of 459 and 201 cases, respectively. The characteristics of GNB patients in the training and testing cohorts were similar to those in the total cohort (Table 2). A new validation cohort was also collected, and the baseline information was also shown in Table 2. The characteristics of the new validation cohort in the survival and non-survival patients were similar to the training and testing cohort.

**Table 2:** Characteristics of training and validation cohort.

Characteristics	Training cohort		*P*-value	Testing cohort		*P*-value	New validation cohort		*P*-value
	Survival	Non-survival		Survival	Non-survival		Survival	Non-survival	
Age (median, IQR, years)	57.5 (49.0–66.0)	58.0 (50.0–64.0)	0.580	54.0 (48.0–64.0)	64.0 (48.50–71.0)	0.128	59.0 (54.0–68.0)	61.0 (52.0–67.5)	0.756
**Gender (n,%)**									
Male	170 (42.3%)	36 (63.2%)	0.005	60 (33.9%)	11 (45.8%)	0.271	42 (43.8%)	10 (52.6%)	0.647
Female	232 (57.7%)	21 (36.8%)		117 (66.1%)	13(54.2%)		54 (56.2%)	9(47.4%)	
**Underlying disease (n,%)**									
Gynecologic cancer	140 (34.8%)	7 (12.3%)	0.001	65 (36.5%)	5 (20.8%)	0.243	30 (31.3%)	2 (10.3%)	0.118
Upper gastrointestinal cancer	42 (10.4%)	10 (17.5%)	0.174	15 (8.4%)	4 (17.4%)	0.315	13 (13.5%)	7 (36.8%)	0.034
Hepatobiliary cancer	41 (10.2%)	7 (12.3%)	0.803	13 (7.3%)	2 (8.7%)	1.000	15 (15.6%)	3 (15.8%)	1.000
Genitourinary cancer	41 (10.2%)	2 (3.5%)	0.168	14 (7.9%)	3 (13.0%)	0.659	15 (15.6%)	1 (5.3%)	0.407
Head and neck cancer	30 (7.5%)	7 (12.3%)	0.322	13 (7.3%)	1 (4.3%)	0.929	6 (6.3%)	2 (10.3%)	0.860
Lung and bronchus cancer	25 (6.2%)	10 (17.5%)	0.006	12 (6.7%)	2 (8.7%)	1.000	1 (1.0%)	1 (5.3%)	0.745
Breast cancer	20 (5.0%)	2 (3.5%)	0.878	16 (9.0%)	1 (4.3%)	0.723	3 (3.1%)	0 (0.0%)	1.000
Other cancers	63 (15.7%)	12 (21.2%)	0.505	29 (16.4%)	6 (25.0%)	0.829	13 (13.5%)	3 (15.8%)	1.000
**Primary infection (n,%)**									
Bloodstream	187 (46.5%)	14 (24.6%)	0.139	94 (53.1%)	8 (28.6%)	0.192	23 (24.0%)	5 (26.3%)	1.000
Pulmonary	69 (17.2%)	27 (47.4%)	< 0.001	21 (11.8%)	10 (35.7%)	< 0.001	13 (13.5%)	5 (26.3%)	0.292
Urinary tract	59 (14.7%)	6 (10.5%)	0.523	30 (16.9%)	2 (7.1%)	0.482	25 (26.0%)	3 (15.8%)	0.510
intraperitoneal infection	33 (8.2%)	9 (15.8%)	0.107	16 (9.0%)	4 (14.3%)	0.370	17 (17.7%)	3 (15.8%)	1.000
Catheter related bloodstream infection	18 (4.5%)	0 (0.0%)	0.206	5 (2.8%)	0 (0.0%)	0.918	6 (6.3%)	0 (0.0%)	0.579
Soft tissue	12 (3.0%)	0 (0.0%)	0.38	4 (2.2%)	1 (3.6%)	1.000	5 (5.2%)	0 (0.0%)	0.688
Biliary tract	24 (6.0%)	1 (1.8%)	0.317	7 (3.9%)	3 (10.7%)	0.167	7 (7.3%)	3 (15.8%)	0.450

Based on multivariate Cox proportional hazards regression analyses, five independent prognostic factors were identified in the training cohort, which were shown in Fig. 2B. The five factors were shock (HR = 4.625, *P* < 0.001), admission to ICU before infection (HR = 3.060, *P* < 0.001), pulmonary infection (HR = 2.316, *P* = 0.003), higher lymphocyte counts (HR = 1.512, *P* = 0.007) and lower PLT counts (HR = 0.993, *P* < 0.001), respectively. The prognostic model was constructed with the five factors. For each outcome, coefficients and hazard ratios (HRs) were calculated, and the coefficients were used to weight each factors of the model. The formula of the model dispayed as follows:

$$\begin{gathered} {\text{h}}\left( {{3}0 {\text{days}}} \right)\, = \,{\text{h}}0\left( {{3}0 {\text{days}}} \right)*{\text{exp}}({1}.{1183}*{\text{ICU}}\, + \,0.{8398}*{\text{pulmonary}}.{\text{infection}} \hfill \\ + \,{1}.{5314}*{\text{shock}}\, + \,0.{4131}*{\text{lymphocyte }} - 0.00{72}*{\text{ PLT}}). \hfill \\ \end{gathered}$$

h(30 days) presented the 30-day survival probability of malignant patients with GNB; h0(30 days) was a constant; ICU represented admission to ICU before infection; pulmonary.infection represented primary infection before GNB was pulmonary infection; shock represented that the patients got shock after GNB; lymphocyte represented lymphocyte counts (*10^9^/L), and PLT represented PLT counts (*10^9^/L).

### Estimation of the prognostic model

**Figure 3:**
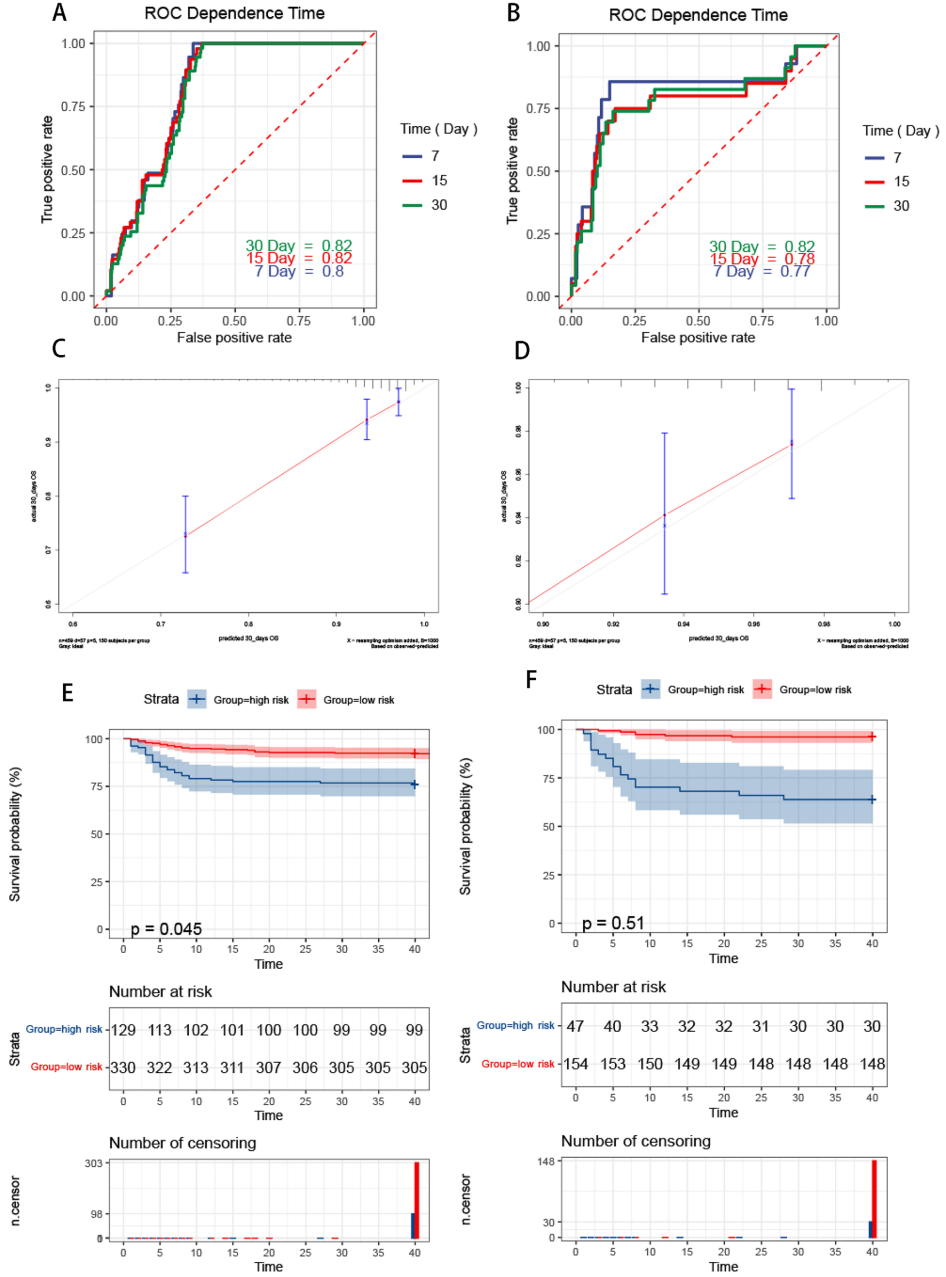
Estimation of the model. ROC curve in training cohort (**A**). ROC curve in testing cohort (**B**). Time-dependent ROC curve analysis of survival prediction by the prognostic model. Calibration curves in training cohort (**C**). Calibration curves in testing cohort (**D**). The Y-axis represents actual survival, as measured by K–M analysis, and the X-axis represents the model-predicted survival. Survival analysis of patients with GNB in the training (**E**) and testing (**F**) sets. The K–M survival curves show the overall survival based on the high and low-risk patients divided by the optimal cut‐off point.

According to the survival probabilities calculated by the model, the time ROC was used to evaluate the diagnostic value of death caused by GNB. The AUCs for the 7-days, 15-days and 30-days were 0.80, 0.82 and 0.82 in the training cohort, respectively. And in the testing cohort, the AUC values of the ROC projected the7-days, 15-days and 30-days were 0.77, 0.78 and 0.82, respectively (Fig. 3A and B). The calibration curve indicated a good agreement between the actual observations and predictions model using the model in both training cohort (Fig. 3C) and the testing cohort (Fig. 3D).

The optimal cut-off value to discriminate nonsurvivors from survivors was 0.929 according to the 30-day ROC curves. The two cohorts were separated into high-risk and low-risk groups based on the probability of 0.929. The Kaplan–Meier analysis was performed to evaluate patients’ OS in the two groups. The results showed that patients in the high-risk group had shorter OS (*P* < 0.001), indicating a significant unfavorable outcome for high-risk GNB (Fig. 3E and F).

**Figure 4:**
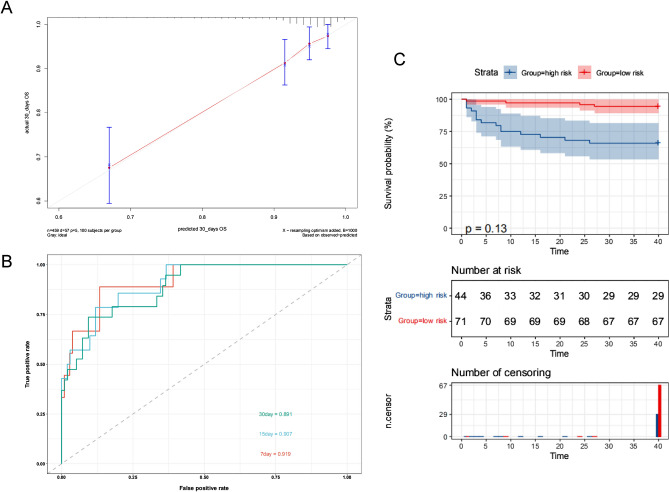
Estimation of the model applying in the new validation cohort. (**A**) Calibration curves. (**B**) ROC curves. (**C**) The K–M survival curves show the overall survival based on the high and low-risk patients divided by the optimal cut‐off point.

In the new validation cohort, the calibration curves also displayed high consistency in the prediction of GNB’s survival time (Fig. 4A). The AUCs for the 7-days, 15-days, and 30-days were 0.91, 0.90, and 0.89 in the new validation cohort (Fig. 4B), which suggested the good prediction capability of this model. The new validation was separated into high-risk and low-risk groups based on the probability of 0.929 as previously. The Kaplan–Meier analysis was performed to evaluate patients’ OS in the two groups. The results showed that patients in the high-risk group had shorter OS (*P* < 0.001) (Fig. 4C).

In order to validate this model in malignant patients with suspected GNB, we collected 50 GNB patients (NB), another 50 fever patients who were proved to be gram-positive bacteremia (PB), and 50 fever patients who were proved to be with no bloodstream infection (NonB). The basic information of the 150 individuals were supplied in Table S4. The AUCs for the 7-days and 30-days were 0.87, and 0.82 in this validation cohort, the sensitivity for the 7-days and 30-days were 0.65 and 0.87 and the specificity for the 7-days and 30-days were 1.00 and 0.64, respectively. These AUCs in independent PB and NonB also suggested a good prediction of this model (Table S5).

### Development of a web server presenting the prognostic nomogram

**Figure 5:**
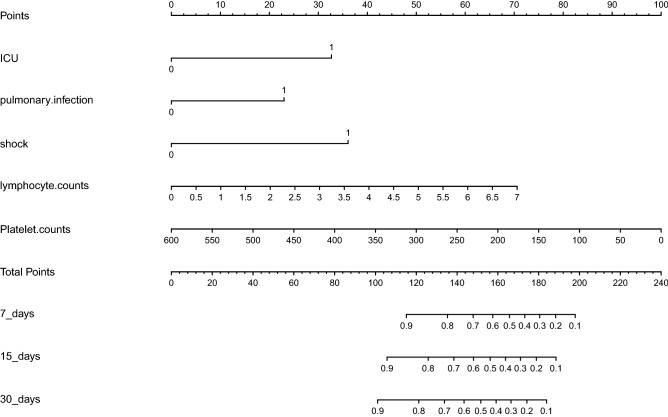
Nomogram predicting mortality in malignant patients with GNB. The nomogram was applied by adding up the points identified on the points scale for each variable. The total points projected on the bottom scales indicate the probabilities of 7-days, 15-days and 30-days OS. ICU represented admission to ICU before infection; pulmonary.infection meant that primary infection before GNB was pulmonary infection; shock meant that the patients got shock after GNB; Lymphocyte. counts meant lymphocyte counts(*10^9^/L); and Platelet. Counts meant platelet counts(*10^9^/L).

Based on the prognostic model, a nomogram predicting 30-day survival probabilities in all the patients with GNB was generated. An online version of our nomogram (Fig. 5) could be accessed*,* which could help clinicians and patients easier access our new model. Predicted survival probabilities across time could be easily determined by inputting clinical and laboratory features, while the reading output figures and tables were also generated by the webserver. The website was shown in the supplementary file.

## Discussion

As the mortality in malignant patients remains high, bloodstream infections is a common, deadly, and costly complications^14^. And gram-negative bacilli was the most frequent bacteria cause of BSIs in malignant patients^15,16^. But there were still very little researches on the risk factors and outcomes of malignant patients with GNB. With cancers, due to the surgery and radiochemotherapy, there might be non-inflammatory fever and changes in some lab results. And most cancers were in the chronic station, which might lead the malignant patients to survive with cancer for several years. A model constructed with the information of malignant patients could be more specific for malignant patients. So we devoted ourselves to finding the risk factors for the mortality in those patients, and integrated these factors as a prognostic model, which could provide evidence for the clinicians making a decision.

In our research, the GNB took about 69.3% of all BSI patients with cancers, the same as previous research, which meant GNB has become an important cause of BSI in patients with cancers^17^. The risk factors for GNB infection in our analysis were also similar to those reported in the previous publications^6,18^. The patients with gynecologic cancer, hepatobiliary cancer, and genitourinary cancer were more likely to get GNB. In gynecologic cancer surgery, the prolonged use of surgical drains was a risk factor for surgical site infection. And gynecologic cancer patients’ fecal carriage of bacteria might increase the risk of bloodstream infections^19^. For these patients, active surveillance of gram-negative bacilli was proved to be an effective strategy to limit the occurrence of GNB in hospital. Postoperative mortality and morbidity rates after hepatobiliary–pancreatic surgery remained high, and enterobacteriaceae were the most common microorganisms that were isolated from these patients. So for hepatobiliary cancer patients, these findings highlighted the importance of safe patient care practices, and the importance of preventing infection^20^. PCT and CRP were thought associated with BSI. Serum PCT concentrations were higher in patients with GNB than in patients with Gram-positive bacteremia or candidemia^21^. Whereas, CRP proved useless in predicting bacteremia, which was similar to our study^22,23^.

The 30-day mortality for GNB was 12.27% in this real-world cohort, which was similar to the prior report^24^. We found shock, admission to ICU before infection, pulmonary infection, higher lymphocyte, and lower PLT were independently associated with high mortality in patients with GNB. The sepsis and sepsis shock always came along with the GNB occurrence, while sepsis shock could lead to higher mortality. The pathogenesis of sepsis shock involves many complex cellulars and biochemical interactions between leukocytes, platelets, endothelial cells, and the complement system that triggered an inflammatory response leading to multi-organic failure^25^. Organ dysfunction and the attendant complications of treating the organ dysfunction lead to a high risk of morbid complications and death^26^. Admission to the ICU in the cancer population was associated with high mortality and did not result in benefit from subsequent cancer treatment^27^. Multidrug-resistant organisms on patient's hands in an ICU setting could be one of the reasons^28^. Most gram-negative bacilli produced necrotizing bronchopneumonia with hemorrhage and abscess formation. Some virulent gram-negative species, such as klebsiella, lead to necrosis, bacteremia, and shock with a propensity to infect the pulmonary microvasculature^29^. Lower platelet counts always reflected poor nutrition and immunity, which indicated the patients had a higher risk of GNB and a poor prognosis^30^. As proved in our study, malignant patients with GNB in the death group had lower platelet counts than patients in the survival group.

As reported, the 180-day mortality rate due to septic shock was higher in cancer patients compared with non-cancer patient^2^. APECHII and SOFA were widely used in the evaluation of infectious shock but were limited for the complex calculation and subjective estimation. So a prognostic model was required to estimate the dead risk of the malignant patients with suspicious GNB more objectively and fastly. Nomograms have previously been widely used in the oncology literature to help patients evaluate the risk of disease progression and mortality^31,32^ For that, a model to predict 30-day mortality in patients with GNB was constructed with a common clinic and laboratory features and presented with online nomogram. No matter applied in training or testing cohort, even a new validation cohort, the model performed well in time ROC. And all the data in this research were real-world data with no intervention from the researchers, which could provide more reliable evidence for this model. The factors used to construct the prognostic model were the results when the blood culture samples were delivered to the laboratory. So the model could be used when the malignant patients were suspicious as GNB and retrieved blood culture samples. If the patients have the high-risk factors for the GNB indicated in our research, they could be evaluated by the model. As the factors were accessible and objective, the dead risk could be evaluated more fleetly and reliably using a nomogram or web tool than APECHII and SOFA. If a high risk was hinted at, the clinicians could intervene more soon. Therefore, we hope this tool could help the clinicians avoid inappropriate treatment and control clinical indicators which were influential in mortality earlier. Combining with novel molecular and phenotypic rapid tests for identification might show potential for favorable influences on patients’ outcomes^33^. Early goal-directed therapy provided significant benefits to outcomes in patients with severe GNB^34^. However, the population used in the model construction and validation might limit the model application. In future work, more malignant patients with suspicious BSI would be further followed, which could validate the existing model in other populations, and provide a larger sample size to construct a new model for more application.

## Conclusion

In conclusion, we have described risk factors for incidence and mortality of GNB in malignant patients based on real-world data, which could provide an accurate and generalizable assessment of the key risk factors for infection and subsequent patient outcomes. Based on our findings, further researches could focus on the risk of morbidity and mortality for specific cancer. Additionally, Sichuan cancer hospital as the Cancer Control and Prevention Center in Sichuan province provided a large number of malignant patients, which made this model more reliable for the malignant patients. But external validation with a prospective cohort is still required, which has been an area of planned future study. To our knowledge, this is the first study to configure a nomogram to predict 30-day mortality in malignant patients with GNB. And the model was presented on the website (Supplementary file)*,* which could be widely used by the clinicians freely.

## Supplementary Information

Supplementary Information.

## Data Availability

The authors declare that all data generated or analyzed for this study are available within the paper and its supplementary information. Additional raw data are available from the corresponding author upon reasonable request.

## Abbreviations

GNB: Gram-negative bacteremia
OS: Overall survival
BSI: Blood stream infection
PCT: Procalcitonin concentration
CRP: C-reactive protein
IQR: Interquartile range
OR: Odds ratio
CI: Confidence interval
HR: Hazard ratio
CRBSI: Catheter-related bloodstream infection
