# Supplementary material for: A novel prognostic model for malignant patients with Gram-negative bacteremia based on real-world research
Source: Sci Rep. 2022 Jul 8;12:11644. doi: 10.1038/s41598-022-15126-5 (PMC9270414; doi:10.1038/s41598-022-15126-5)
Supplement: Supplementary file 1 — Supplementary Information. [file 41598_2022_15126_MOESM1_ESM.docx]

**A novel prognostic model for malignant patients with Gram-negative bacteremia based on real-world research**

**Supplementary file**

**Sujiao Ni ^1,#^, Pingyao Xu^1,#^, Kaijiong Zhang^1^, Haiming Zou^1^, Huaichao Luo ^1^, Chang Liu ^1^, Yuping Li ^1^, Yan Li ^2^, Dongsheng Wang** **^1*^, Renfei Zhang^3*^, Ruiling Zu^1^**^*^

^1^ Department of Clinical Laboratory, Sichuan Cancer Hospital & Institute, Sichuan Cancer Center, School of Medicine, University of Electronic Science and Technology of China, Chengdu, Sichuan, China

^2^ Chengdu University of Traditional Chinese Medicine, Chengdu, Sichuan, China

^3^Department of Clinical Laboratory, The Third Hospital of Mianyang(Sichuan mental health center), Mianyang, Sichuan, China

**Model website:**

https://cancer-patients-gram-negative-bacteremia-death-predictor.shinyapps.io/DynNomapp/?_ga=2.239628352.2112552927.1650121905-244268814.1649916801

**Figures and Tables**

**1. Figure S1. The comparisons of laboratory features between GNB (+) and GNB (-) groups. (A) The concentration of C reaction protein. (B) procalcitonin concentration. (C) white blood cell counts. (D) neutrophil counts. (E) lymphocyte counts. (F) platelet counts. (G) Mean platelet volume. (H) Platelet distribution width.**

**
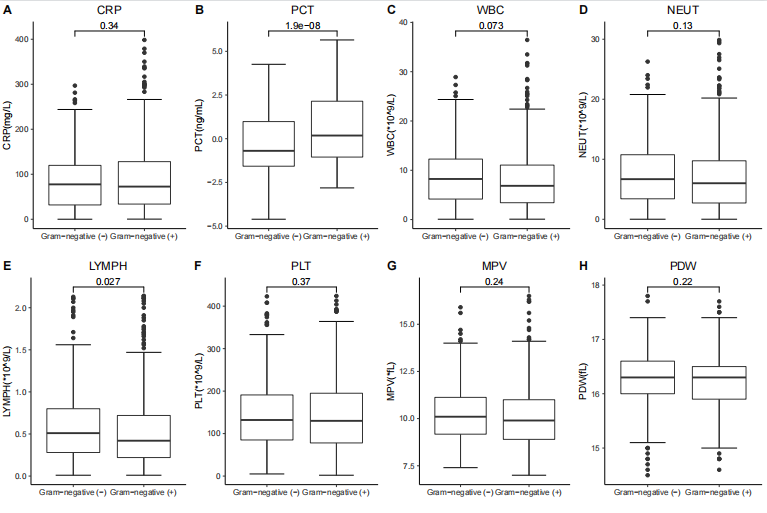
**

**2. Figure S2 Forest plots of mortality at 30 days for malignant patients with GNB.**


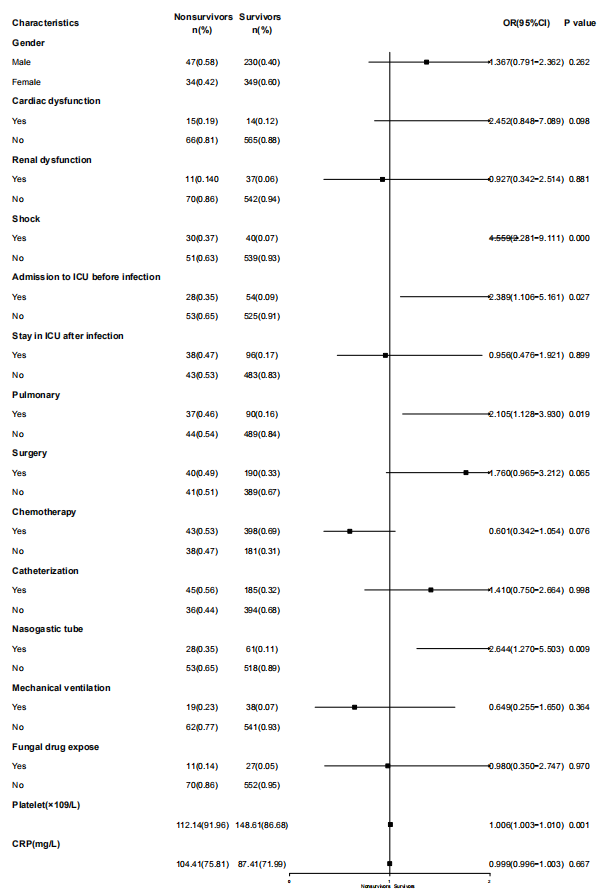


3. The prognostic model could be accessed at the website: https://cancer-patients-gram-negative-bacteremia-death-predictor.shinyapps.io/DynNomapp/?_ga=2.239628352.2112552927.1650121905-244268814.1649916801.

The variables could be chosen in the bar chart, and a predicted survival probability and survival plot could be presented after clicked “predict” button.

4. **Table S1 The comparisons of risk factors between Gram-negative bacilli infection group (GNB (+)) and non-Gram-negative bacilli infection group (GNB (-)).**

| **Characteristics** | **GNB (+) ( n=660)** | **GNB (-) (n=344)** | **P-value** |
| --- | --- | --- | --- |
| Age (median, IQR, years) | 57.0(49.0-66.0) | 59(48.0-67.25) | 0.101 |
| **Gender (n, %)** |  |  | 0.000 |
| Male | 277(42.0%) | 189(54.9%) |  |
| Female | 383(58.0%) | 155(45.1%) |  |
| **Underlying disease (n, %)** |  |  |  |
| Gynecologic cancer | 217(32.9%) | 59(17.2%) | 0.000 |
| Upper gastrointestinal cancer | 71(10.8%） | 53(15.4%) | 0.034 |
| Hepatobiliary cancer | 63（9.5%） | 14(4.1%) | 0.002 |
| Genitourinary cancer | 60(9.1%) | 19(5.5%) | 0.046 |
| Head and neck cancer | 51(7.7%) | 46（13.4%） | 0.004 |
| Lung and bronchus cancer | 49(7.4%) | 50（14.5%） | 0.000 |
| Breast cancer | 39(5.9%) | 22（6.4%） | 0.760 |
| Hematologic cancer | 39(5.9%) | 39（11.3%） | 0.002 |
| Lower gastrointestinal cancer | 31(4.7%) | 25（7.3%） | 0.092 |
| pancreatic cancer | 22(3.3%) | 9（2.6%） | 0.533 |
| other cancers | 18(2.7%) | 8（2.3%） | 0.704 |
| **Co-morbidities (n, %)** |  |  |  |
| diabetes | 66(10.0%) | 41(11.9%) | 0.350 |
| hypertension | 98(14.8%) | 58(16.9%) | 0.404 |
| chronic obstructive pulmonary disease | 14(2.1%) | 15(4.4%) | 0.044 |
| cardiac dysfunction | 29(4.4%) | 23(6.7%) | 0.120 |
| hapatic dysfunction | 131(19.8%) | 67(19.5%) | 0.888 |
| renal dysfunction | 48(7.3%) | 34(9.9%) | 0.152 |
| **infection status (n, %)** |  |  |  |
| neutropenic | 17(2.6%) | 4(1.2%) | 0.138 |
| shock | 70(10.6%) | 45(13.1%) | 0.243 |
| multiple pathogens | 18(2.7%) | 13(3.8%) | 0.361 |
| nosocomial infection | 560(84.8%) | 294(85.5%) | 0.795 |
| admission to ICU before infection | 82(12.4%) | 66(19.2%) | 0.004 |
| length of hospital staying before infection(>3days) | 597(90.5%) | 312(90.7%) | 0.901 |
| **primary infection (n, %)** |  |  |  |
| Bloodstream | 307(46.5%) | 151(43.9%) | 0.429 |
| pulmonary | 127(19.2%) | 108(31.4%) | 0.000 |
| urinary tract | 97(14.7%) | 28(8.1%) | 0.003 |
| intraperitoneal infection | 62(9.4%) | 24(7.0%) | 0.194 |
| Catheter related bloodstream infection | 23(3.5%) | 21(6.1%) | 0.054 |
| soft tissue | 17(2.6%) | 19(5.5%) | 0.017 |
| biliary tract | 35(5.3%) | 7(2.0%) | 0.014 |
| **treat status (n, %)** |  |  |  |
| surgery | 230(34.8%) | 101(29.4%) | 0.072 |
| hormone | 439(66.5%) | 217(63.1%) | 0.278 |
| immunosuppressive | 39(5.9%) | 22(6.4%) | 0.760 |
| chemotherapy | 441(66.8%) | 223(64.8%) | 0.527 |
| parenteral nutrition | 27(4.1%) | 22(6.4%) | 0.108 |
| central venous catheter | 449(68.0%) | 243(70.6%) | 0.397 |
| catheterization | 230(34.8%) | 108(31.4%) | 0.272 |
| nasogastric tube | 89(13.5%) | 58(16.9%) | 0.151 |
| mechanical ventilation | 57(8.6%) | 41(11.9%) | 0.096 |
| antibiotic expose | 279(42.3%) | 173(50.3%) | 0.015 |
| fungal drug expose | 38(5.8%) | 25(7.3%) | 0.349 |

**5. Table S2 Multivariable analysis for GNB independent risk factors**

| Characteristics | OR (95%CI) | P-value |
| --- | --- | --- |
| Gender | 0.712(0.505-1.004) | 0.053 |
| Gynecologic cancer | 1.634(1.034-2.582) | 0.036 |
| Upper gastrointestinal cancer | 1.002(0.609-1.650) | 0.993 |
| Hepatobiliary cancer | 2.382(1.209-4.692) | 0.012 |
| Genitourinary cancer | 2.112（1.096-4.067） | 0.025 |
| Head and neck cancer | 0.763（0.455-1.281） | 0.307 |
| Lung and bronchus cancer | 0.649（0.380-1.110） | 0.012 |
| Hematologic cancer | 0.568（0.327-0.986） | 0.044 |
| chronic obstructive pulmonary disease | 0.699（0.315-1.554） | 0.379 |
| admission to ICU before infection | 0.876（0.582-1.318） | 0.525 |
| pulmonary | 0.642（0.452-0.913） | 0.014 |
| urinary tract | 1.283（0.778-2.117） | 0.329 |
| soft tissue | 0.437（0.213-0.893） | 0.023 |
| biliary tract | 1.953（0.810-4.709） | 0.136 |
| antibiotic expose | 0.801（0.595-1.078） | 0.144 |
| PCT | 0.967（0.951-0.983） | 0.000 |
| LYMPH | 1.166（0.932-1.460） | 0.179 |
| Catheter related bloodstream infection | 0.443（0.231-0.847） | 0.014 |

6. **Table S3 The comparisons of mortality risk factors between survivors and non-survivors in GNB group.**

| **Characteristics** | **Non-survivors  (n=81)** | **Survivors  (n=579)** | **P-value** |
| --- | --- | --- | --- |
| Age (median, IQR, years) | 62(50.0-66.0) | 57(49.0-65.0) | 0.176 |
| **Gender (n, %)** |  |  |  |
| Male | 47(58.0%) | 230(39.7%) | 0.002 |
| Female | 34(42.0%) | 349(60.3%) |  |
| **Co-morbidities (n, %)** |  |  |  |
| diabetes | 9(11.1%) | 57(9.8%) | 0.722 |
| hypertension | 15(18.5%) | 83(14.3%) | 0.322 |
| cardiac dysfunction | 15(18.5%) | 14(2.4%) | 0.000 |
| hapatic dysfunction | 19(23.5%) | 112(19.3) | 0.385 |
| renal dysfunction | 11(13.6%) | 37(6.4%) | 0.020 |
| **infection status (n, %)** |  |  |  |
| shock | 30(37.0%) | 40(6.9%) | 0.000 |
| nosocomial infection | 74(91.4%) | 486(83.9%) | 0.081 |
| admission to ICU before infection | 28(34.6%) | 54(9.3%) | 0.000 |
| stay in ICU after infection | 38(46.9%) | 96(16.6%) | 0.000 |
| length of hospital staying before infection (>3days) | 75(92.6%) | 522(90.2%) | 0.485 |
| **primary infection (n, %)** |  |  |  |
| Bloodstream | 30(37.0%) | 277(47.8%) | 0.068 |
| pulmonary | 37（5.7%) | 90(15.5%) | 0.000 |
| urinary tract | 8(9.9%) | 89(15.4%) | 0.191 |
| intraperitoneal infection | 12(14.8%) | 50(8.6%) | 0.074 |
| surgery | 40(49.4%) | 190(32.8%) | 0.004 |
| hormone | 48(59.3%) | 391(67.5%) | 0.140 |
| chemotherapy | 43(53.1%) | 398(68.7%) | 0.005 |
| central venous catheter | 56(69.1%) | 393(67.9%) | 0.820 |
| catheterization | 45(55.6%) | 185(32.0%) | 0.000 |
| nasogastric tube | 2834.6%) | 61(10.5%) | 0.000 |
| mechanical ventilation | 19(23.5%) | 38(6.6%) | 0.000 |
| antibiotic expose | 39(48.1%) | 240(41.5%) | 0.253 |
| fungal drug expose | 11(13.6%) | 27(4.7%) | 0.001 |
| **Laboratory test results** |  |  |  |
| WBC (median, IQR, ×109/L） | 7.95(3.35-11.81) | 6.75(3.45-10.93) | 0.356 |
| Platelet (median, IQR, ×109/L) | 95(44.0-158.0) | 136.0(83.0-200.5) | 0.000 |
| MPV (median, IQR, fL) | 10.10(9.20-11.50) | 9.80(8.80-10.90) | 0.115 |
| PDW (median, IQR, fL) | 16.40(16.0-16.70) | 16.20(15.90-16.50) | 0.015 |
| CRP (median, IQR, mg/L) | 100.23(48.39-145.42) | 70.18(32.44-125.61) | 0.030 |
| PCT (median, IQR, ng/mL) | 3.54(0.99-18.05) | 0.98(0.32-7.23) | 0.000 |
| NEUT (median, IQR, ×10^9^/L） | 7.23(2.21-10.17) | 5.96(2.75-9.50) | 0.032 |
| LYMPH (median, IQR, ×10^9^/L） | 0.40(0.21-0.84) | 0.42(0.22-0.73) | 0.742 |

1. In order to validate this model in malignant patients with suspected GNB, we collected 50 GNB patients (NB), another 50 fever patients who were proved to be gram-positive bacteremia (PB), and 50 fever patients who were proved to be with no bloodstream infection (NonB). The basic information of NB, PB, NonB and suspected GNB (including NB, PB, and NonB) were shown in **Table S4.** The model was evaluated in NB, PB, NonB and suspected GNB using ROC curves. The ROC curves could reflect the sensitivity (Sens), specificity (Spec), and the area under the curve (AUC), which showed in **Table S5.**

**Table S4 Basic information of suspected GNB**

| **Characteristics** | **NB (n=50)** | **PB (n=50)** | **NonB (n=50)** | **suspected GNB (n=150)** |
| --- | --- | --- | --- | --- |
| Age (median, IQR） | 60.0(54.50-67.75) | 58.50(46.50-66.75) | 61.0(50.50-69.0) | 59.0(50.0-68.0) |
| **Gender** |  |  |  |  |
| Male | 23(46.0%) | 29(58.0%) | 34(68.0%0 | 86(57.3%) |
| Female | 27(54.0%) | 21(42.0%) | 26(52.0%) | 64(42.7%) |
| ICU | 15(30.0%) | 6(12.0%) | 26(52.0%) | 47(31.3%0 |
| Pulmonary infection | 7(14.0%) | 13(26.0%) | 2346.0%) | 43(28.7%) |
| shock | 10(20.0%) | 4(8.0%) | 3(6.0%0 | 17(11.3%) |
| Lymphocyte (median, IQR） | 0.35(0.23-0.61) | 0.39(0.25-0.73) | 0.67(0.36-1.20) | 0.45(0.28-0.86) |
| PLT (median, IQR） | 139.50(82.25-198.75) | 130.50(71.25-177.75) | 182.0(115.5-305.0) | 154.0(92.0-201.8) |
| 7days death | 4(8.0%) | 3(6.0%) | 2(4.0%) | 9(6.0%) |
| 30 days death | 7(14.0%) | 8(16.0%) | 7(14.0%) | 22(14.7%) |

**Table S5 Performance parameters of the model in malignant patients with suspected GNB**

| **Class** | **Performance parameter** | **Value** |
| --- | --- | --- |
| NB-7days | Sensitivity | 1.00 |
|  | Specificity | 0.75 |
|  | AUC | 0.93 (0.79, 1.07) |
| NB-30days | Sensitivity | 0.63 |
|  | Specificity | 1.00 |
|  | AUC | 0.86 (0.72, 1.00) |
| PB-7days | Sensitivity | 0.65 |
|  | Specificity | 1.00 |
|  | AUC | 0.78 (0.58, 0.98) |
| PB-30days | Sensitivity | 0.69 |
|  | Specificity | 0.75 |
|  | AUC | 0.77 (0.59, 0.95) |
| NonB-7days | Sensitivity | 0.88 |
|  | Specificity | 1.00 |
|  | Accuracy | 0.91 (0.81, 1.00) |
| NonB-30days | Sensitivity | 0.86 |
|  | Specificity | 0.86 |
|  | AUC | 0.88 (0.72, 1.03) |
| NB+PB+NonB-7days | Sensitivity | 0.65 |
|  | Specificity | 1,00 |
|  | AUC | 0.87 (0.76, 0.97) |
| NB+PB+NonB-30days | Sensitivity | 0.86 |
|  | Specificity | 0.64 |
|  | AUC | 0.82 (0.73, 0.91) |
